# Duodenal somatostatinoma: a case report and review of the literature

**DOI:** 10.1186/1752-1947-7-115

**Published:** 2013-04-25

**Authors:** Oguz Koc, Yigit Duzkoylu, Yavuz Selim Sari, Hasan Bektas, Gungor Uzum, Vahit Tunali, Esra Pasaoglu

**Affiliations:** 1 General Surgery Department, Istanbul Education and Research Hospital, Org. Abdurrahman Nafiz Gurman Street, Fatih Istanbul, 34096, Turkey; 2 Pathology Department, Istanbul Education and Research Hospital, Org. Abdurrahman Nafiz Gurman Street, Fatih Istanbul, 34096, Turkey

**Keywords:** Neuroendocrine, Pancreaticoduodenectomy, Somatostatinoma

## Abstract

**Introduction:**

About 70% of well-differentiated endocrine tumors arise from the gastrointestinal tract. Duodenal well-differentiated endocrine tumors account for only 2.6% of all neuroendocrine tumors. Following the first two case reports of somatostatin-secreting tumors in 1977, fewer than 200 cases of somatostatinoma have been reported. These tumors of the duodenum are usually silent and asymptomatic, but can cause gastrointestinal symptoms. Depending on the localization of the tumor, multiple surgical procedures can be performed, ranging from local resection to pancreaticoduodenectomy.

**Case presentation:**

Here, we report a case of a submucosal duodenal mass in a 42-year-old Turkish White man presenting with nausea, vomiting, fatigue and abdominal pain. The treatment decision of pancreaticoduodenectomy made preoperatively was later altered to intraoperative removal via local resection with sphincteroplasty.

**Conclusion:**

Tumors of the periampullary region are considered highly malignant, and the Whipple operation is usually the only procedural treatment. In the current case, we decided not to perform pancreaticoduodenectomy but to excise the mass intraoperatively, and consequently avoided unnecessary resection of the pancreas and anastomosis to undilated hepatic and pancreatic ducts. This protective strategy prevented duodenum- and pancreas-related morbidity.

## Introduction

Carcinoid tumors of the duodenum are usually hormonally silent endocrine cell tumors. Langhans is often credited with the first description of a gut carcinoid tumor in 1867 [1]. Gastroenteropancreatic endocrine tumors are rare neoplasms, with one to four cases documented per 100,000 people each year [2]. Since the first two case reports of somatostatin-secreting tumors in 1977, fewer than 200 cases of somatostatinoma have been reported [3]. Classification of neuroendocrine tumors (NETs) of the duodenum is based on hormone production, for instance, carcinoid (serotonin), somatostatinoma, or gastrinoma, because each induces a distinct clinical syndrome. About 70% of well-differentiated endocrine tumors (WDET) arise from the gastrointestinal tract [4]. Duodenal WDETs account for only 2.6% of all NETs [5,6]. Ileal NETs and/or carcinoids are frequently located in the small bowel (>70%), but recent studies have shown that NETs of the duodenum are more commonly encountered (22%) [7]. Somatostatin-producing tumors also give rise to the somatostatin syndrome, which was initially reported by Krejs *et al*. in 1979 [8]. Typical somatostatin syndrome comprises the triad of diabetes mellitus, cholelithiasis, and steatorrhea [8]. These tumors can be excised using invasive or noninvasive procedures, depending on their localization.

In our case, because histopathologic findings were insufficient and unconfirmed preoperatively, a pancreaticoduodenectomy was planned, but after intraoperative exploration, the mass was thought to be a NET and resected locally, with frozen section examination showing malignancy-free margins. Neuroendocrine cell tumors should always be considered if found in the periampullary region, as in our case. Here, we have reported a case of nonfunctional duodenal somatostatinoma showing regional lymph node and sphincter of Oddi metastases, and reviewed the literature focusing on the latest clinical outcomes of this disease.

## Case presentation

**Figure 1:**
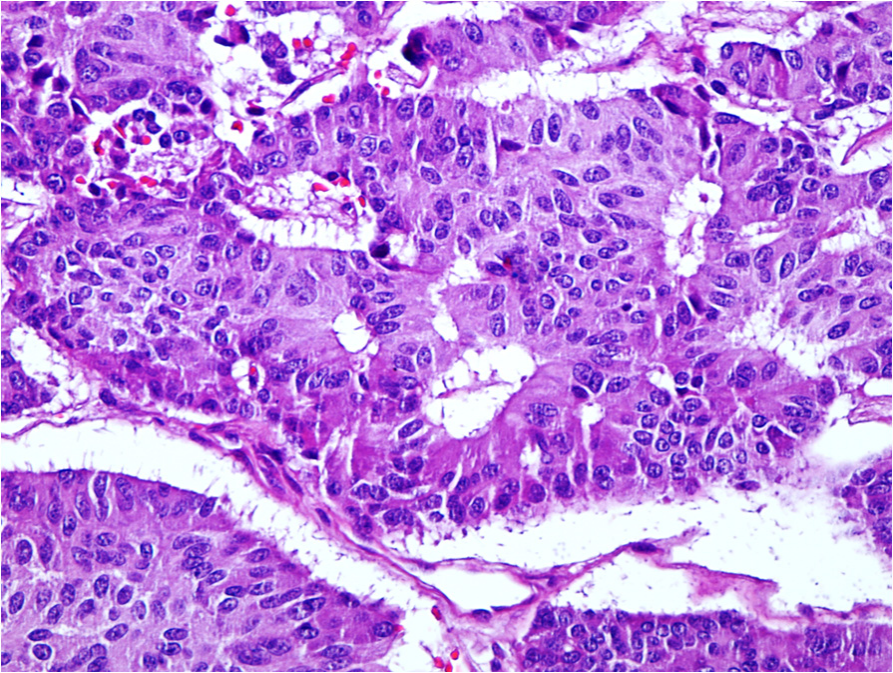
Image of the pathological examination with paraffin embedding and staining.

The patient was a 42-year-old Turkish White man presenting with nausea, vomiting, fatigue and abdominal pain. Laboratory data revealed no abnormalities in blood chemistry or tumor markers. An enzyme-linked immunosorbent assay disclosed positivity for HBsAg. No abnormal findings were observed in the chest X-ray. Computed tomography revealed thickening at the second and third portions of the duodenal wall and a polypoid contrast-enhanced area. Because a gastroduodenoscopy showed no abnormalities, an endoscopic retrograde cholangiopancreatography (ERCP) was performed. The ERCP revealed a duodenal mass. Biopsy specimens derived by ERCP were highly suspicious for malignancy. Subsequent color Doppler examination performed for abdominal aorta and portal vein invasion did not reveal any abnormalities. The plan was to perform a pancreaticoduodenectomy, and 10 days later the patient underwent surgery. A mobile, polypoid, palpable tumor was further uncovered in the third portion of the duodenum, distal to the duodenal papilla. Pancreas, papilla and duodenal wall except the third portion seemed to be free from the polypoid mass. Considering the possibility of a NET tumor, the relatively invasive procedure was postponed, complete tumoral tissue was excised locally, and the specimen sent for frozen section examination. Pathological examination revealed that resected tumor margins were free of malignancy. However, owing to the proximity of the tumor to the ampulla of Vater, a sphincteroplasty was conducted to prevent potential obstruction. In addition, cholecystectomy and choledochotomy procedures were performed, and a T-tube inserted into the choledochus. Finally, all the enlarged lymph nodes were resected. Pathological examination with paraffin embedding and staining disclosed a 1.7cm well-differentiated, Grade 1 neuroendocrine carcinoma (NEC) (Figure 1). Tumor cells were detected in the muscularis propria of the sphincter of Oddi. Lymphovascular invasion of tumor cells (a total of eight lymph nodes) was positive. According to the TNM staging system, the tumor was classified as pT2. The tumor consisted of uniform cells formed in glandular patterns. Tumor cells were stained for chromogranin A, synaptophysin and somatostatin. The final diagnosis was made by the pathology unit as primary duodenal somatostatinoma. The proliferation index was high and because of the lymphovascular invasion, the tumor was thought to be malignant. Because the patient had been operated on for papillary thyroid cancer 6 years earlier, the possibility of multiple endocrine neoplasia was excluded. The level of neuron-specific enolase was 14.4ng/mL at the second postoperative week. The patient was discharged from the hospital with no complications on postoperative day 22.

## Discussion

Carcinoid tumors of the duodenum are generally low-grade NETs. The term ‘neuroendocrine’ is derived based on similarities between these cells and neural cells in terms of expression of specific proteins, such as synaptophysin, neuron-specific enolase and chromogranin A [9]. These tumors arise from enterochromaffin cells of the gastrointestinal tract. The gastrointestinal tract contains at least 14 different endocrine cell types, including enterochromaffin cells that are classically associated with carcinoid tumors [10]. In the duodenum, most NETs are nonfunctional, sporadic, well differentiated and slow growing [11]. Ultimately, 22% of all NETs of the small bowel arise from the duodenum [7]. Duodenal carcinoids are most common in the proximal duodenum and present as early enhancing intraluminal polyps or mural masses [12]. The symptoms usually consist of abdominal complaints, such as pain, nausea, vomiting and bleeding. The majority of patients do not display distant organ metastases. For duodenal somatostatinomas, the somatostatin syndrome can occur only if the tumor is larger than 4cm [13]. Somatostatin is a cyclic tetradecapeptide secreted by the hypothalamus, cerebrum, spinal cord, vagus nerve, and D cells in Langerhans islets of the pancreas, stomach, duodenum, and small intestine.

In our patient, the tumor originated from the third portion of the duodenum and was measured as 1.7cm. The patient presented with nonspecific gastrointestinal symptoms, such as abdominal pain, fatigue, nausea and vomiting. The serum insulin, glucagon, gastrin, somatostatin and urine 5-hydroxyindoleacetic acid levels were not assessed because the mass was not considered a NET preoperatively. Histopathologically, a characteristic structure is the psammoma body, which has been identified in 68% of duodenal somatostatinomas [14]. On silver staining, the majority of carcinoid tumors are argyrophilic, but seldom argentaffinic [15]. The tumor characteristics of our patient were consistent with those of somatostatinoma. Gastrointestinal NETs appear at all ages, with the highest incidence recorded from the fifth decade upwards [16]. NETs of the stomach, duodenum, appendix or rectum are small (≤1cm) and well differentiated; they are considered ‘early’ tumors because they generally have very good prognosis [17]. In the new World Health Organization classification of 2010, these neoplasms are grouped as grade 1 or 2 NETs and/or carcinoids (NETs), and distinguished from poorly differentiated NECs, G3. Duodenal carcinoids are usually detected at the early and treatable stages as a result of considerable progress in the latest imaging techniques. Using these techniques, even very small lesions can be diagnosed. The general widespread use of gastrointestinal endoscopy has led to a shift in the discovery of smaller (≤10 to 20mm) gastrointestinal NETs and/or carcinoids at the time of diagnosis [17]. Endoscopic ultrasound examination is highly sensitive for detecting NETs of the duodenum because it facilitates the identification of submucosal lesions and staging [18].

Primary surgical resection of the tumor and regional lymph nodes is the only curative treatment for gastrointestinal NETs. This is usually possible in about 20% of patients [18]. There is a wide consensus that surgery should be recommended for tumors larger than 20mm. Unfortunately, opinion on the optimal extent of surgical treatment is divided, and therapeutic options for NETs of the duodenum that are nonfunctional, well differentiated and 10 to 20mm in size are controversial. The impact of regional lymph node metastases on survival is uncertain at present. Regardless of the primary tumor size, if abnormal lymph nodes are detected, the tumor and all the regional lymph nodes should be removed. Burke *et al*. [19] identified three pathologic features of the primary tumor as independent risk factors for metastasis: invasion of the muscularis propria, size greater than 2cm, and the presence of mitotic figures. Soga [20] showed that lymph node metastasis is associated with 9.8% of gastrointestinal NETs with submucosal invasion, even in cases when the tumor diameter is 1.0cm or less, suggesting that the risk of metastasis does not differ appreciably from that of carcinoma. In terms of duodenal carcinoids, even small lesions are associated with a risk of lymph node metastasis, which is related to tumor diameter and depth of invasion. In general, carcinoid tumors arising from the small intestine have the greatest propensity to metastasize, even at small sizes, compared with those in the appendix, colon and rectum [21]. Adjuvant chemotherapy for somatostatinomas is not recommended owing to insufficient data, and palliative chemotherapy for unresectable or advanced tumors has limited benefits.

NETs of the small bowel are increasing in incidence. With access to new diagnostic and imaging techniques, detection of these tumors in the United States of America has increased by 300% to 500% in the past 35 years. Simultaneously, prognosis of these tumors has improved considerably [17]. Data from the United States of America have revealed that the incidence of gastrointestinal NETs has increased at a rate of 3% to 10% per year over the past three decades [6,22]. The overall 5-year survival rate for patients with gastrointestinal NETs and/or carcinoids has improved by almost 20% in the last 35 years [6]. The overall 5-year survival rate for patients with somatostatinoma is 40% to 60% [1], 40% in somatostatinomas with liver metastasis, and 100% in tumors without liver or lymph node metastases [23].

Tumors of the periampullary region are considered highly malignant, and the Whipple operation is usually the only procedural treatment. This procedure is a pancreaticoduodenectomy, which is fairly invasive with a number of postoperative potential complications, such as pancreatic fistula, gastroparesis, and malabsorption. Overall survival after Whipple operation for pancreatic adenocarcinoma is about 20% at 5 years after surgery. However, the optimal resection extent of duodenal tumors has not been defined as yet. A number of researchers advocate pancreaticoduodenectomy [24] for all patients with malignant tumors of the duodenum, including those located in the third and fourth portions, to ensure adequate *en bloc* resection. If the tumor is an adenocarcinoma or carcinoid, *en bloc* resection and systemic lymph node dissection are indicated [25]. Others support pancreaticoduodenectomy for proximal duodenal carcinoma, but segmental resection for tumors in the third and fourth portions [26-28]. In the current case, we decided not to perform pancreaticoduodenectomy but to excise the mass intraoperatively, and consequently avoided unnecessary resection of the pancreas and anastomosis to undilated hepatic and pancreatic ducts. This protective strategy prevented duodenum- and pancreas-related morbidity.

## Conclusions

Tumors of the periampullary region are considered highly malignant, and the Whipple operation is usually the only procedural treatment. In the current case, we decided not to perform pancreaticoduodenectomy but instead to excise the mass intraoperatively, and consequently avoided unnecessary resection of the pancreas and anastomosis to undilated hepatic and pancreatic ducts. This protective strategy prevented duodenum- and pancreas-related morbidity.

## Consent

Written informed consent was obtained from the patient for publication of this case report and any accompanying images. A copy of the written consent is available for review by the Editor-in-Chief of this journal.

## Competing interests

The authors declare that they have no competing interests.

## Authors’ contributions

OK and YSS were major contributors in writing the manuscript. YD evaluated the literature and statistical data. HB, GU, and VT analyzed and interpreted the patient data. EP performed the histopathological examination. All authors read and approved the final manuscript.
